# Analysis of the operation of on farm emergency slaughter of bovine animals in the Republic of Ireland

**DOI:** 10.1186/s13620-016-0063-8

**Published:** 2016-03-15

**Authors:** Paul McDermott, Aideen McKevitt

**Affiliations:** 1 Veterinary Department, Mayo County Council, Dublin, Ireland; 2 College of Agriculture, Food Science and Veterinary Medicine, University College Dublin, Dublin, Ireland; 3 Ulster University, School of Biomedical Sciences, Coleraine, Northern Ireland

**Keywords:** On Farm Emergency Slaughter, Casualty Slaughter, *Survey Monkey*, PVPs, OVs

## Abstract

**Background:**

On Farm Emergency Slaughter (OFES) is the slaughter outside the slaughterhouse, of an otherwise healthy animal, which has suffered an accident that, for welfare reasons, prevented its transport to a slaughterhouse. The procedure is designed to prevent the transport of welfare compromised animals, which may have veterinary certification to slaughterhouses for Casualty Slaughter (CS), and provides an alternative to the euthanasia and disposal of injured animals that are otherwise fit for human consumption. The aim of this study was to analyse the operation of OFES in the Republic of Ireland between 1st January 2011 and 31st December 2013.

**Methods:**

Data were obtained from the Animal Identification and Movement electronic database of the Department of Agriculture, Food and the Marine. Two structured surveys were designed, one for Official Veterinarians (OVs) who work in slaughterhouses and the second for Private Veterinary Practitioners (PVPs) who work in food animal practice in the Republic of Ireland. Surveys were administered through *SurveyMonkey*. The total number of bovines slaughtered and the number that underwent OFES in Northern Ireland and the Netherlands were obtained from the Northern Ireland Department of Agriculture and Rural Development and the Netherlands Food and Consumer Safety Authority.

**Results:**

OFES is neither widely available nor used in the Republic of Ireland. Results from the OV survey showed that Food Business Operators consider that facilitation of OFES would be detrimental to business. Data from the 5 slaughterhouses which offer OFES showed that acceptance criteria are not standardised. Results from the PVP survey showed that 77 % (*n* = 79) of PVPs were willing to certify animals for OFES. Fifty four percent (*n* = 49) were aware of slaughterhouses in their area that provided the service of OFES and 64 % (*n* = 57) stated a willingness to certify the transport of acutely injured animals to slaughterhouses for CS. Data from the Northern Ireland Department of Agriculture and Rural Development and the Netherlands Food and Consumer Safety Authority indicated a low level of uptake of OFES in the Republic of Ireland compared to Northern Ireland and the Netherlands.

**Conclusion:**

Based on results reported here, criteria for assessment of risk associated with accepting animals for OFES should be reconsidered. A review of the systems pertaining to OFES and its implementation should be undertaken, including the level and quality of training of all stakeholders, with a view to making OFES more widely available in the Republic of Ireland.

## Background

On Farm Emergency Slaughter (OFES) is the slaughter outside the slaughterhouse, of an otherwise healthy animal that has suffered an accident and which for animal welfare reasons, has prevented its transport to a slaughterhouse. Casualty Slaughter (CS) is the slaughter at a slaughterhouse, of an injured animal that has been deemed fit for transport under veterinary certification [22]. OFES of animals for human consumption has been ratified in the Republic of Ireland since 2009 [5]. Prior to its introduction, the only alternative for acutely injured animals was euthanasia, or transport of injured animals to the slaughterhouse for CS. A study conducted in the Republic of Ireland between 2006 and 2008, demonstrated that of bovine cases consigned under veterinary certification to four large slaughterhouses, over 60 % of the animals could have been designated OFES, if the procedure had been available [18, 19]. In 2008 the Food and Veterinary Office of the European Commission advised the Irish Competent Authority, the Department of Agriculture, Food and the Marine that it was to ensure that animals not fit for transport were slaughtered in situ as required by European Council Directive 93/119 [10, 24]. The Department of Agriculture, Food and the Marine complied in 2009 with the introduction of S.I. 373 of 2009 [5], thus implementing the requirements of Regulation (EC) No 853/2004 of The European Parliament and The Council [11].

OFES requires all the inspection parameters of normal slaughter such as ante-mortem examination and post-mortem examination, with additional food safety and food quality checks since OFES animals are injured [21]. No prescriptive method exists on how OFES should be performed however outline requirements are given in legislation and guidelines [7, 8, 11, 22].

The Department of Agriculture, Food and the Marine and Local Authorities oversee the operation of OFES and both have operational procedures to which Official Veterinarians (OVs), Private Veterinary Practitioners (PVPs) and Food Business Operators must adhere [4, 23]. For OFES to occur, Food Business Operators must firstly allow OFES animals into their slaughterhouses for processing. After ante-mortem examination by the PVP and if the animal is deemed suitable for OFES, a consultation takes place between the PVP and the OV. If accepted, OFES is performed, typically on farm, by either a PVP, or a slaughter person with a certificate of competence for slaughter [9]. Slaughter must take place in compliance with Council Regulation (EC) No1099/2009 [14]. All parts of the carcass including the blood must be brought to the slaughterhouse in a suitable vehicle.

Dressing of carcasses must take place immediately (within 30 min) after intake to the slaughterhouse and a post-mortem examination is carried out as soon as possible after dressing [11, 12]. OFES carcasses are sampled for antibiotic residues, a mandatory procedure, and animals over 48 months are tested for Bovine Spongiform Encephalopathy [15]. Since 1st July 2014 with the introduction of Commission Regulation (EU) No 218 of 2014, meat from OFES animals can be sold across the European Union. Previously meat from an OFES animal had to remain in the member state in which it was slaughtered [16].

The aim of this study was to analyse and evaluate the availability and operation of OFES and CS in the Republic of Ireland in the period between 1st January 2011 and 31st December 2013, and to compare the findings with the situation in Northern Ireland and the Netherlands, two jurisdictions where OFES data were made readily available to the authors. Current practices, including criteria for acceptance by the OV, procedures in relation to OFES and attitudes of stakeholders to OFES were also investigated.

## Methods

Data on the number of OFES and CS animals processed in the Republic of Ireland between 1st January 2011 and 31st December 2013 and the locations where processing took place were obtained from the Animal Identification and Movement electronic database of the Department of Agriculture Food and the Marine [1].

A questionnaire based survey (the OV survey) was designed in the online survey software *SurveyMonkey* and sent by e-mail to all OVs involved in food safety (*n* = 100).

A second survey (the PVP survey) was designed and forwarded to 601 members of the Food Animal Group of Veterinary Ireland, the national representative group for Veterinarians in the Republic of Ireland, using their data base VetALERT (*n* = 601). Members work in food animal practice in Ireland and a number also work as Temporary Veterinary Inspectors in slaughterhouses. Mostly multiple choice and Likert scale questions were used. There were however opportunities for each sample group to include their own opinion. Each survey was piloted on the respective target group and amended where necessary.

Data on total number of bovines slaughtered and number of bovines slaughtered as OFES in Northern Ireland and the Netherlands from 1st January 2011 to 31st December 2013 were obtained from the Northern Ireland Department of Agriculture and Rural Development and the Dutch Food and Consumer Safety Authority, respectively. Figures for CS animals are not available for either Northern Ireland or the Netherlands.

Online survey data were collected between 4th and 23rd June 2014. The online survey data were automatically uploaded into Microsoft Excel. Statistical analysis was conducted using SPSS v21.0. Descriptive statistics were generated for both sets of survey data. Chi-squared tests were used to analyse for statistically significant differences between OFES and CS. Statistical significance was set at a probability of less than 5 % i.e. *p* < 0.05. Ethical approval was obtained from the research ethics committee at Ulster University. A letter was sent to each group of participants assuring confidentiality and each response was coded to ensure data protection.

## Results

Thirty Department of Agriculture, Food and the Marine slaughterhouses and 198 Local Authority slaughterhouses were approved for bovine slaughter in the Republic of Ireland and according to the Animal Identification and Movement electronic database. Of these, 3 Department of Agriculture, Food and the Marine slaughterhouses and 6 Local Authority slaughterhouses accepted OFES carcases between 1st January 2011 and 31st December 2013. Only 6 of the 9 slaughterhouses consistently provided the OFES service during this period. For example, 1 slaughterhouse processed 1 OFES carcase between 2011–2013, while another processed 176 OFES carcases in 2012 but none during 2011 or during 2013 (Table 1).

**Table 1:** Total Slaughter, Casualty Slaughter and On Farm Emergency Slaughter associated with Department of Agriculture Food and the Marine and Local Authority Slaughterhouses between 2011 and 2013 as reported by the Animal Identification Movement electronic database of the Department of Agriculture, Food and the Marine

	Total Slaughter		OFES		Casualty Slaughter	
Year	DAFM	LA	DAFM	LA	DAFM	LA
2011	1,570,323	72,935	69	32	334	1
2012	1,400,858	83,054	119	206	275	10
2013	1,499,551	88,754	71	59	260	11
Total	4,470,732	244,743	259	297	869	22

A total of 94.5 % (*n* = 4,470,732) of all bovine slaughter in the Republic of Ireland during 2011–2013 occurred in Department of Agriculture, Food and the Marine slaughterhouses and 5.5 % (*n* = 244,743) in Local Authority slaughterhouses. During the same period, Department of Agriculture, Food and the Marine slaughterhouses accounted for 97.5 % (*n* = 869) of CS animals and Local Authority slaughterhouses accounted for 2.5 % (*n* = 22). In terms of OFES, 46.6 % (*n* = 259) were processed in Department of Agriculture Food and the Marine slaughterhouses while 54.4 % (*n* = 297) were processed in Local Authority slaughterhouses. During the years in question a significantly greater proportion of OFES was associated with Local Authority slaughterhouses and a significantly greater proportion of CS was associated with Department of Agriculture, Food and the Marine slaughterhouses (*p* < 0.05).

**Fig. 1:**
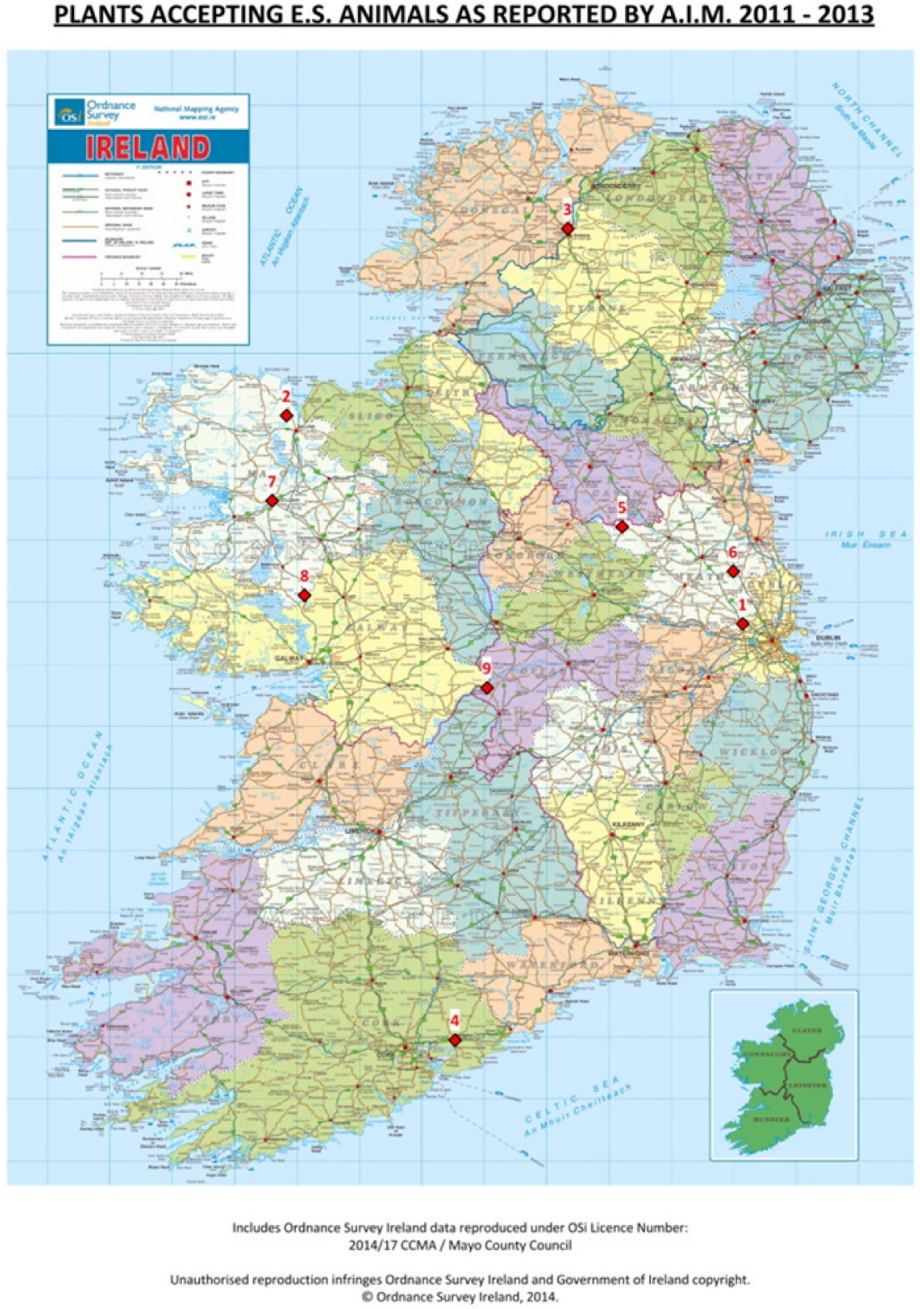
Geographic distribution of 9 Slaughterhouses accepting On Farm Emergency Slaughtered animals between 2011–2013 as reported by the Animal Identification & Movement Electronic Database of the Department of Agriculture, Food and the Marine

The geographic spread of the slaughterhouses (Fig. 1) processing OFES animals highlights the limited availability of the procedure to farmers and PVPs. Slaughterhouses were located in Mayo (3), Meath (3), Cork (1), Donegal (1) and Offaly (1). In contrast, 23 Department of Agriculture Food and the Marine slaughterhouses, with a wide geographical spread, accepted CS animals in the same period.

### Survey of official veterinarians

The results of the survey administered to OVs are shown in Table 2.

**Table 2:** Responses to Official Veterinarian (*n* = 46) survey in relation to acceptance of On Farm Emergency Slaughtered and Casualty Slaughtered animals under Veterinary Certification into Slaughterhouses

Responses	Numbers
Food Business Operators did not accept OFES animals.	41(89 %)
Food Business Operators accepted OFES animals.	5(11 %)
Food Business Operators perceived that OFES would have a negative impact on consumer perception of their business.	28(61 %)
Official Veterinarians accepted animals for CS with veterinary certification.	23(50 %)
Official Veterinarians did not accept animals for CS with veterinary certification..	9(20 %)
OVs perceived there was an increased food safety risk to consumers from consuming OFES meat.	8(17 %)
OVs perceived there was an increased risk of Dark, Firm and Dry meat and a decrease in quality	7(15 %)

A majority 89 % (*n* = 41) of Food Business Operators were not in favour of accepting OFES carcases into their premises, for business reasons. OVs who responded to the survey, stated that in terms of OFES, there is an increased food safety risk to consumers from consuming OFES meat 17 % (*n* = 8), and an increased risk of Dark, Firm and Dry meat and therefore a decrease in quality 15 % (*n* = 7). Fifty percent (*n* = 23) of OVs who responded stated that they had accepted animals with a veterinary certificate for casualty slaughter. Five OVs oversaw slaughterhouses that accepted OFES carcases. Their responses to survey questions were inconsistent in terms of acceptance criteria, procedures and practices. These results are shown in Table 3.

**Table 3:** Acceptance Criteria, Procedures and Practices in 5 Slaughterhouses that accepted OFES animals, (OV Responses *n* = 5)

Responses	Numbers
OV always insisted on a consultation with PVP following ante-mortem examination	5/5
OV/Temporary Veterinary Inspector were always present at intake of OFES animal into slaughterhouse	5/5
OV always sampled for antibiotics	4/5
OV/Temporary Veterinary Inspector were always present at dressing of carcase	3/5
Licensed Slaughterman always performed slaughter	3/5
OV/Temporary Veterinary Inspector were always present at dressing of carcase	3/5
OV accepted animals with open fractures for OFES	2/5
Slaughterhouses had age restrictions due to the non availability of Bovine Spongiform Encephalopathy testing	2/5
Slaughterhouse had weight restriction due to abattoir capacity	2/5
Meat from OFES carcases always returned to the farm of origin	2/5
PVP performed slaughter occasionally and Primary Producer transported animal	1/5
PVP performed slaughter always and Primary Producer transported animal	1/5
Slaughterhouse had chilled transport for transporting OFES animals	1/5

All OVs insisted on a consultation with a PVP following ante-mortem examination and that an OV/Temporary Veterinary Inspector was always present at OFES intake. However, two OVs accepted animals with open fractures, two imposed age restrictions, two imposed weight restrictions and two insisted that OFES meat return to the farm of origin.

### Survey of private veterinary practitioners

Ninety responses (15 %) were obtained to the survey sent to the Food Animal Group of Veterinary Ireland. These results are shown in Table 4.

**Table 4:** PVP Responses (*n* = 90) to Survey on Procedures, Practices and Clinical Conditions Pertaining to OFES

Response	Numbers
Very likely/likely to have recommend OFES to their clients	79(88 %)
Very Familiar/Familiar with Farm Animal Welfare Advisory Council Guidelines	76(84 %)
Agreed with the OFES of animals with open fractures	67(74 %)
Strongly agreed/agreed with consultation between OV and PVP	56(62 %)
Agreed with OFES of animals with an inconclusive diagnosis, but deemed fit for human consumption	51(52 %)
Aware of slaughterhouses in your area that provided the service of OFES	49(54 %)
Agreed with OFES of downer animals, but fit for human consumption	44(50 %)
Aware that a consultation took place between the OV and PVP following ante-mortem examination	43(48 %)
Agreed with the transport of acutely injured animals under certain circumstances to slaughterhouses for CS	32(36 %)
Agreed with the transport of acutely injured animals for CS in any circumstance	25(28 %)
Agreed with the OFES of animals injured longer than 48 h but fit for human consumption	16(18 %)
PVP performs OFES at all times	2(2 %)

As shown in Table 4, a majority of respondents, 88 % (*n* = 79), stated that they were likely to recommend OFES to their clients but only slightly more than half, 54 % (*n* = 49), were aware of slaughterhouses in their area that provided the service. A majority, 62 % (*n* = 56), agreed that a consultation should take place between the OV and PVP following the ante-mortem examination but only 48 % (*n* = 43) were aware that a consultation did take place. A majority of PVP respondents, 84 % (*n* = 76), stated that they were familiar with the Food Animal Welfare Advisory Council Guidelines on Management of Acutely Injured Animals [22].

A majority of respondents 74 % (*n* = 67) agreed with the OFES of animals with open fractures. Approximately one quarter 28 % (*n* = 25) of respondents agreed with the transport of acutely injured animals under any circumstance and 36 % (*n* = 32) agreed with transport of acutely injured animals under certain circumstances. In terms of ranking procedures, when presented with an acutely injured animal, PVPs ranked OFES, treatment, CS and euthanasia in that order.

When asked about criteria for allowing transport of acutely injured animals under veterinary certification for CS, responses of PVPs are shown in Table 5.

**Table 5:** PVPs responses (*n* = 32) to circumstances in which PVPs would allow the transport of animals for CS under Veterinary Certification

Responses	Numbers
Transport of animal would not entail further suffering	11(26 %)
Transport time or distance was short	11(26 %)
Animal could walk onto trailer	5(12 %)
Animals were left waiting too long for OFES, CS performed faster	5(12 %)

### Results from Northern Ireland Department of agriculture and rural development and the Netherlands food and consumer safety authority

Figures obtained from the Northern Ireland Department of Agriculture and Rural Development showed that 0.11 % (*n* = 3,657) of bovine animals slaughtered underwent OFES and from the Food and Consumer Safety Authority in the Netherlands the figure was 0.90 %. (*n* = 13,497).

## Discussion

We analysed the operation of OFES and CS in the Republic of Ireland between 1st January 2011 and 31st December 2013 using the available data from Animal Identification and Movement electronic database of the Department of Agriculture, Food and the Marine. Results showed that CS significantly outweighed OFES and that the majority of CS occurred at Department of Agriculture, Food and the Marine slaughterhouses despite the introduction of legislation in 2009 to ensure that animals not fit for transport were to be slaughtered in situ. This research is the first analysis of the implementation of OFES since the legislation was introduced.

According to Cullinane et al. [18] over 60 % of CS certified animals could have been certified for OFES had the facility been available. The situation has improved somewhat since the introduction of the OFES legislation in that 39 % of acutely injured animals which previously may have been consigned to CS underwent OFES during the period 2011–2013.

However, in large areas of the Republic of Ireland there is as yet no OFES service and even where provided it is inconsistently applied. Between 2011 and 2013, Cork in the south of Ireland which has 15 % of the national bovine herd, had only 1 OFES animal, while Mayo in the west of Ireland which has 4 % of the national herd, accounted for 140 OFES animals during the same period. OFES is virtually unavailable south of a line drawn from Galway to Dublin. Comments from the surveys about the limited availability of OFES included that an OFES service is expensive and some slaughterhouses provide it only for *privileged clients*, i.e. producers who slaughter large numbers of animals.

According to the OV survey, slaughterhouses that did provide for OFES adhered to Standard Operating Procedures but there were differences in acceptance criteria between slaughterhouses. No set criteria for what is acceptable by OVs for OFES were reported. Some OVs accepted open fractures, some had age and weight restrictions and some insisted on meat returning to the farm of origin. All penetrating skin wounds associated with fractures must be considered to be infected because of the severity of contamination with dirt and manure typical of food animals [3]. A mixed bacterial flora can usually be cultured from a swab sample taken from a wound [25, 26]. A risk assessment should be carried out by the Competent Authority to ascertain whether animals with open wounds constitute a food safety risk to the consumer.

Another acceptance criterion that should be assessed is the period of time that defines the acute phase of injury. Research indicates that 48 h is a reasonable period of time to associate with the acute phase of injury [20].

Results from the OV survey indicated that sampling for antibiotic residues which is mandatory, is not always performed. There may be a justification for the targeted testing of OFES animals for anthelminthics [17].

Results from the OV survey indicated that some Food Business Operators had made a business decision not to allow OFES carcases into their slaughterhouses for a variety of reasons including negative perception of the business, financial margins that would not justify the work involved, and that meat quality from some OFES animals renders it unmarketable. For these reasons, some Food Business Operators have insisted that meat from OFES be returned to the farm of origin. However, since 1st July 2014, meat from OFES animals is not required to remain on the home market, and this may have a positive effect on the number of slaughterhouses providing for OFES by increasing the customer base and also because processing may be less logistically problematic.

A large majority of PVPs were aware of Farm Animal Welfare Advisory Council guidelines and therefore aware of OFES, however only 54 %, were aware of slaughterhouses providing the OFES service in their area. This highlighted the lack of availability of OFES. The restrictive criteria for acceptance associated with OFES was given as the reason by some PVPs for not recommending OFES to clients. There was a discrepancy between what PVPs considered to be acceptable criteria for OFES and what OVs deemed as acceptable criteria for admittance into slaughterhouses. This may indicate that PVPs were not fully aware of the risks to consumers from OFES meat while OVs were more risk averse. A number of PVP respondents reported that they had recommended OFES to clients more than 7 times indicating that where the service is available, and where PVPs were aware of it, they were willing to certify animals for OFES. The very low level of OFES performed exclusively by PVPs indicates that the majority of OFES was performed by a licensed slaughter person working in a slaughterhouse that provides the service. The PVP survey results indicate that a majority of PVPs in the Republic of Ireland did not have the equipment or expertise necessary to carry out OFES. These results suggest that further training is required for PVPs if OFES is to become more widely available. This is in contrast to the situation in Northern Ireland where PVPs do carry out OFES regularly.

OFES was reported as the first ranked option by PVPs when presented with an acutely injured animal, whose injury renders it unfit for transport, but availability of OFES limits the use of this option. CS was reported as the third ranked option after treatment. However treatment may not be an option due to the nature and severity of an injury and the degree of pain experienced by an animal. Veterinary Ireland has published a policy document which outlines how pain should be managed [6]. A majority of PVP respondents agreed with the transport of animals for CS under certain circumstances such as transport over a short distance/time. Veterinarians should be aware that in certifying an animal for CS, they are certifying that the transport of an acutely injured animal will not cause further unnecessary suffering and if transport would cause further unnecessary suffering, then the only legal alternatives are OFES or euthanasia and disposal. In some cases the nature and severity of an injury was not the determining factor in the choice of CS or OFES, rather it was the availability of OFES or CS that determined which procedure took place. Additionally, some OVs and PVPs considered that OFES and CS are not mutually exclusive. Regulation (EC) 1 of 2005 sets out the rules in relation to fitness of animals for transport. It states that no animal shall be transported unless it is fit for the intended journey. However sick or injured animals may be considered fit for transport if transport would not cause additional suffering [13].

The results from the PVP survey regarding acceptance of animals with an inconclusive diagnosis for OFES, and acceptance of downer animals by approximately 50 % of respondents are interesting considering that both issues encompass a range of conditions that could render an animal unfit for human consumption. These responses warrant further investigation into the understanding by PVPs, of the requirements for veterinary certification at ante-mortem examination.

Results from the Netherlands Food and Consumer Safety Authority and the Northern Ireland Department of Agriculture and Rural Development indicated that the uptake of OFES in the Republic of Ireland was low compared with the Netherlands and Northern Ireland. In Northern Ireland, PVPs performed the majority of OFES and the farmer arranged for the transport of the carcase to the slaughterhouse. There were no figures available for CS slaughter but under The Welfare of Animals (Transport) Regulations (Northern Ireland) 2006 and associated guidance, the requirement is for farmers/hauliers to consider whether or not an animal is “fit for transport” or to seek a professional opinion from a Veterinarian who will advise on “Fitness for Transport” if asked to do so. In the Netherlands, farmers and transporter are penalised if they transport animals not fit to travel to a slaughterhouse. The Netherlands does not have a system for accepting CS animals into slaughterhouses. In the Republic of Ireland, during the last five years, there has been no finding against any Veterinarian by the fitness to practice committee of The Veterinary Council of Ireland in relation to the certification of acutely injured animals, unfit for transport.

## Conclusion

In conclusion, between 1st January 2011 and 31st December 2013, OFES was provided in 4 % of slaughterhouses and in a limited geographical area in the Republic of Ireland. For OFES to become more widely used it will be necessary to increase engagement with all stakeholders. Results suggest that criteria for the risks associated with acceptance of animals for OFES should be reconsidered, and a risk analysis of these criteria performed. A review of OFES and its implementation, including quality and training of all stakeholders should be undertaken, with a view to making the procedure more widely available. Results from these activities could be used to develop fact sheets about OFES that would be made available to OVs, PVPs, and Food Business Operators and would explain the procedures and risks associated with OFES.

Additionally, a more robust enforcement of the rules governing transport of welfare compromised animals should be undertaken as is the case in the Netherlands. The veterinary profession must ensure that it provides good advice to producers and carries out its responsibilities for animal welfare and consumer protection. The Veterinary Public Health Association in the United Kingdom has stated that the route to achieving greater animal welfare safeguards and consumer protection lies in closer working relations between PVPs and OVs [2].
